# Dicer Deletion in the Ear Can Cut Most Neurons and Their Innervation of Hair Cells to Project to the Ear and the Brainstem

**DOI:** 10.3390/ijms27010539

**Published:** 2026-01-05

**Authors:** Ebenezer N. Yamoah, Gabriela Pavlinkova, Jeong Han Lee, Jennifer Kersigo, Marsha L. Pierce, Bernd Fritzsch

**Affiliations:** 1 Department of Translational Neurosciences, College of Medicine, University of Arizona, Phoenix, AZ 85004, USA; enyamoah@arizona.edu (E.N.Y.); jeongl@arizona.edu (J.H.L.); 2 Institute of Biotechnology of the Czech Academy of Sciences, 25250 Vestec, Czech Republic; gpavlinkova@ibt.cas.cz; 3 Department of Pathology, University of Iowa Health Care, Iowa City, IA 52246, USA; jennifer-kersigo@uiowa.edu; 4 Department of Pharmacology, College of Graduate Studies, Midwestern University, Downers Grove, IL 60515, USA; mpierc1@midwestern.edu; 5 Department of Neurological Sciences, University of Nebraska Medical Center, Omaha, NE 68198, USA

**Keywords:** Dicer, miRNA, neurogenesis, vestibular neurons, cochlear neurons, development, projections, hair cells, vestibular nuclei

## Abstract

Dicer is crucial for the generation of microRNAs (miRNAs), which are essential for regulating gene expression and keeping neuronal health. Dicer’s conditional deletion cuts all spiral ganglion neurons but spares a small fraction of vestibular ganglion neurons, innervating the utricle and part of the saccule. Hair cells develop in the utricle, saccule, posterior crista, and the cochlea in *Pax2^Cre^*; *Dicer^f/f^*. Cochlear hair cells develop at the base and expand the OHC and IHC in the middle, or split into a base/middle and the apex. In contrast, *Foxg1^Cre^*; *Dicer^f/f^* cuts all canal cristae and cochlea hair cells, leaving a reduced utricle and an exceedingly small saccule. Likewise, *Foxg1^Cre^*; *Gata3^f/f^* shows no cochlear hair cells and is absent in the horizontal and reduced in the posterior crista. In contrast, the utricle, saccule, and anterior crista are nearly normal, underscoring the intricate regulatory networks involved in hair cell and neuronal development. The central projections have been described as the topology of various null deletions. Still, without spiral ganglion neurons, fibers from Dicer null mice navigate to the cochlear nuclei and expand into the vestibular nuclei to innervate the caudal brainstem. Beyond a ramification around the CN, no fibers expand to reach the cerebellum, likely due to *Pax2* and *Foxg1* that cut these neurons. Genetic alterations, such as Dicer deletion, can lead to hearing loss and impairments in auditory signal processing, illustrating the critical role of microRNAs in the development and function of auditory and vestibular neurons. Further studies on this topic could help in understanding potential therapeutic targets for hearing loss associated with neuronal degradation of miRNA.

## 1. Introduction

Vertebrate ears extract sound, angular, and linear acceleration from mechanical stimuli and develop a 3D geometry that connects sensory epithelia (hair cells and supporting cells) via sensory neurons to the brainstem. Molecular analysis over the last 25 years has revealed aspects of the molecular basis for patterning, morphogenesis, and histogenesis of the ear and the brainstem [1,2,3]. How they are integrated into a program that ensures the right genes are regulated at the right time and in the correct place remains unclear. Essential factors that translate early patterning processes into specific areas of expression in developing prosensory epithelia, which in turn govern the morphogenesis of semicircular canals, otoconia, and the cochlea, are not fully known [4,5]. Molecular steps in histogenesis of the vestibular and cochlear begin to emerge, indicating that hair cell formation requires the coordinated transition of gene expressions such as *Eya1*, *Sox2*, *Neurog1*, *Isl1*, and *Neurod1* [6,7,8] to eventually upregulate *Atoh1* in postmitotic hair cells to initiate differentiation of these hair cells [2,3]. All of these processes are not only characterized by upregulation of novel gene expression but also by a coordinated downregulation of previously important gene expression, likely involving microRNA (miRNA) to eliminate already existing transcripts to hasten the transition from an expression profile characterizing epithelial cells to another characterizing sensory cells and neurons [9,10,11]. Moreover, previous work showed that the inner ear expresses about 100 miRNAs [12,13], including hair cell-specific miRNAs [14]. Overall, we underscore the complexity of genetic regulation in ear development and the critical roles of signaling pathways and miRNAs in shaping the structure and function of this sensory organ to make hair cells, its vestibular and spiral ganglion neurons, and brainstem nuclei.

miRNAs have a pivotal role in a wide array of plant and animal development and organ functions. Currently, about 1900 miRNAs (see miRbase; releases 22.1) have been catalogued, of which about 500 have been described based on evidence and expression [15,16]. miRNAs require Pol II to start transcription with Drosha and DGCR8 to generate a pre-miRNA that is exported from the nucleus to the cytosol. Dicer is an enzyme that plays a crucial role in the processing of microRNAs and small interfering RNAs (siRNAs). It cleaves long double-stranded RNA (dsRNA) and precursor miRNA (pre-miRNA) molecules into short, functional RNA fragments, which are then incorporated into the RNA-induced silencing complex (RISC) to regulate gene expression [17,18]. Dicer is crucial to generate a specific miRNA population; it dictates the specificity of miRNA of approximately 22–24 bp with a terminal loop and the 3′ overhang [16]. The conserved Dicer (*Dicer-1*) gene encodes a 220 kDa multidomain protein that is dedicated to the miRNA pathway [19]. In the absence of Dicer, no canonical miRNA will be generated and will typically interact with other gene expression to regulate about 60% specific expression [9], except for deviating miRNAs [20]. Haploinsufficiency with only one functional allele shows specific cancer development [21,22]. Moreover, evolution is known across all archaea, bacteria and eucaryotic and has specific duplication in non-vertebrates [23,24] and is present in the common origin of life, LUCA [25,26]. Simple Dicer deletion blocks normal development beyond the earliest mice development [27]. Using conditional deletion of other genes, such as *Pax2^Cre^* or *Foxg1^Cre^*, can eliminate the floxed Dicer and can provide the absence of specific miRNAs and has specific losses of ganglion neurons, hair cells, and brainstem nuclei [28,29,30,31].

There is an emphasis on the need for detailed analyses comparing the effects of Dicer deletions with other gene mutants. We will compare the effect of Dicer deletion in neurons and their peripheral and central projection with other null mutants to provide a detailed analysis of *Shh* [32], *Neurog1* [33], *Neurod1* [6,34], *Pax2* [35,36], *Gata3* [37], *Lmx1a/b* [38], and *Irx3/5* DKO [39]. This will provide insights into the intricate network of genetic interactions that govern the development of sensory systems within the ear and the brainstem [40,41]. Further research in this field could lead to enhanced understanding of auditory and vestibular system disorders and potential therapeutic strategies for miRNAs [10].

## 2. Results

### 2.1. Dicer Deletion Results in the Loss of Most Vestibular Neurons

The sequence of gene expressions in neuronal generation can be tentatively categorized as follows: Eya1 > Neurog1 > Neurod1, among others [2,5,42,43]. In the absence of *Eya1,* there is limited expression of *Neurog1* [42,44]. Specific genes, like *Sox2* and *Isl1*, are needed for the normal development of neurons [41,42]. However, despite a later loss of neurons in *Sox2* deletion [45,46], an initial neuronal formation can be demonstrated, using in situ hybridization (ISH), showing overall expression of *Neurog1*, *Isl1*, and *Neurod1* [42,47]. Likewise, *Neurod1* null deletion was thought to result in the loss of all neurons [48], but a more detailed analysis showed an altered neuronal connection in the ear [6,34,49]. Using *Pax2^Cre^* and *Foxg1^Cre^* to alter inner ear genes shows a more incomplete deletion of *Pax2^Cre^*; *Gata3^f/f^* compared to *Foxg1^Cre^*; *Gata3^f/f^* [37]. Moreover, using antibodies against caspase 3 shows an upregulation of vestibular neurons and eventual loss of nearly all fibers, including dye-tracing [42,47]. Other genes interfere with the normal development of vestibular fiber innervation of hair cells, such as the posterior canal [*Fgf10* [50]] or the horizontal canal [*Foxg1*; [51]] or in the case of Sema, there is an expanded fiber projection [52] similar to the misexpression of *Bdnf*/*Ntf-3* in a transgenic model [53,54]. Moreover, central projection showed the near-normal vestibular fibers in a *Foxg1^Cre^*; *Sox2^f/f^* null mice that lose all central projection in about E16.5 old mice [47].

**Figure 1:**
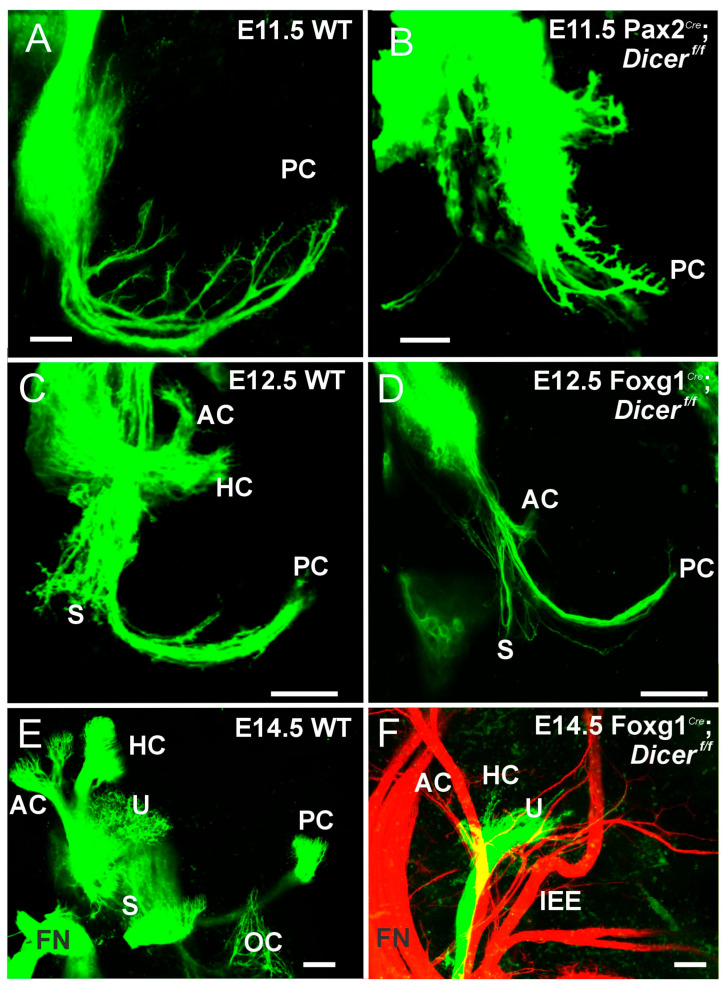
The developmental role of Dicer in the vestibular ear. Control mice develop vestibular ganglion neurons (VGNs) that send out fibers around E11 (**A**). One day later ((**C**), E12.5), the formation of the posterior canal (PC), anterior and horizontal canals (AC, HC), and the saccule (S) is seen. At a later stage (E14.5), the five vestibular projections (AC, HC, U, S, PC) are visible, along with the organ of Corti (OC, (**E**)). Initially, fiber projections are close to wild type (WT) (**A**,**B**), but by E12.5, there is a reduction in fibers extending toward their targets (**C**,**D**). Without Dicer, fibers become restricted to innervating the utricle (U) and have inner ear efferent (IEE, red) that extend toward the AC and HC (**E**,**F**). The facial nerve (FN) wraps around both control and Dicer null mutants (**E**,**F**). Abbreviations: AC, anterior crista; FN, facial nerve; HC, horizontal crista; OC, organ of Corti; PC, posterior crista; S, saccule; U, utricle. Modified from (**B**) [29] using lipophilic dye tracing. Bar shows 100 µm.

**Figure 2:**
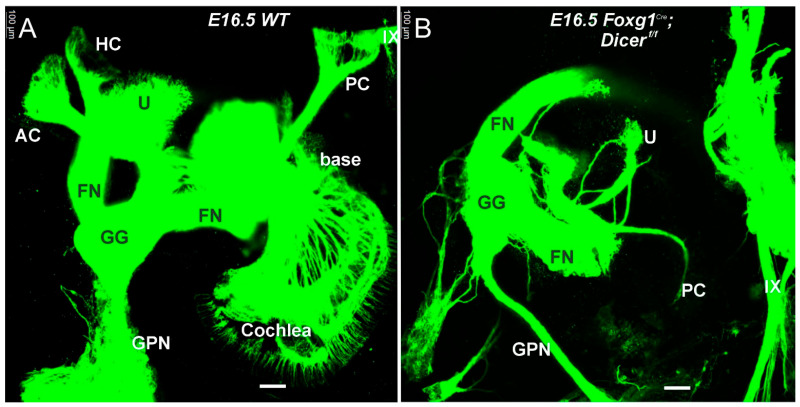
Comparison of Dicer null with WT. This ear was traced by dye injection into the brainstem and was prepared as a whole ear. It leaves the vestibular and cochlear projection that emanates near the facial nerve (FN) to branches to the greater petrosal nerve (GPN) from the geniculate ganglion (GG; (**A**,**B**)). A small, diminished fiber projections extend toward the posterior crista (PC) compared to the control projection (**A**,**B**). There is no extension towards the anterior or posterior crista in *Foxg1^Cre^*; *Dice^f/f^* compared to control (AC, HC; (**A**,**B**)), while a small projection innervates the utricle (U; (**A**,**B**)). A growing cochlear nerve is ventral to the facial nerve (**A**) and is missing in the Dicer null mutant (**B**). Note that the IX cranial nerve is close to the PC in control mice (**A**), while there is no approximation of a PC that labels IX cranial nerve much closer to the smaller ear (**B**). AC, anterior crista; FN, facial nerve; GG, geniculate ganglion; GPN, greater petrosal nerve; HC, horizontal crista; PC, posterior crista; U, utricle; IX, glossopharyngeal nerve. Modified from (**B**) [28] using lipophilic dye tracing. Bar shows 100 µm.

**Figure 3:**
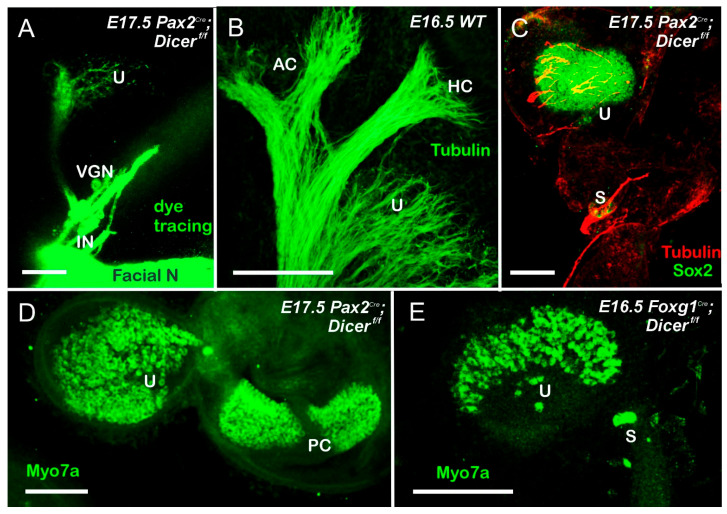
Only a small utricle is present in both conditional Dicer deletions. (**A**,**B**) Dye tracing in the brainstem shows the facial nerve and a small projection to navigate the small utricle (**A**). In contrast, nerve fibers branch to innervate the utricle’s anterior and horizontal cristae. (**B**). A small population of utricular and a microscopic saccular neuron that receives fibers labeled with a tubulin antibody (**C**). (**D**) shows the Myo7a antibody in the posterior crista (PC) compared with the nearby utricle. An even smaller utricle and saccule are present in a *Foxg1^Cre^*; *Dicer^f/f^*. Abbreviations: AC, anterior crista; HC, horizontal crista; IN, intermediate nerve; PC, posterior crista; S, saccule; U, utricle; VGN, vestibular ganglion neurons. Modified from (**A**,**C**–**E**); [28,29] using dye tracing (**A**) or antibody staining (**B**–**E**). Bar shows 100 µm.

Dicer deletion takes 3–10 days to deplete specific miRNA [29] while deletion of *Pax2* or *Foxg1* can eliminate neurons with a shorter delay, ~12–24 h [37]. Even the earliest onset of these Dicer deletions using *Foxg1^Cre^* or *Pax2^Cre^* shows near-normal first neuron development and near-normal projections to the periphery, including the ear (Figure 1). However, the projection of nerve fibers is diminished. It has a limited projection to the ear, as shown by dye tracing or an antibody, innervating the utricle and saccule (Figure 1 and Figure 2). In contrast, the posterior crista forms but does not reach out to innervate the posterior hair cells (Figure 3) in E17.5 old *Pax2^Cre^*; *Dicer^f/f^* [29]. A smaller utricle and saccule (Figure 3) ensue in *Foxg1^Cre^*; *Dicer^f/f^*, which lacks all canal crista formation [28]. Moreover, caspase 3 is upregulated in the VGNs, indicating that the neurons are dying [28,29,30]. Additionally, the smaller ear has reduced otoconia formation in the *Pax2Cre*; *Dicer^f/f^* (Supplemental Figure S1). No otoconia can be found in *Foxg1^Cre^*; *Dicer^f/f^*, which likely requires *Irx3/5* to develop otoconia, tectorial membrane, and cupulae [39]. Meanwhile, alterations in miRNA, specific for miR-124, resulted in nearly absence of ears [29]. In summary, even with a delay in Dicer loss, nearly all VGNs are lost, except for the utricle and a small saccule neuron. There is an absence of the posterior crista that reaches out toward the canal cristae in *Pax2^Cre^*; *Dicer^f/f^*, but there was no hair cell innervation, like in Fgf10 null mice [50], while all canal cristae hair cells and innervation were absent in *Foxg1^Cre^*; *Dicer^f/f^*.

### 2.2. Dicer Requires Spiral Ganglion Neurons

Spiral ganglion neurons (SGNs) develop between E10 and E12.5, progressing from basal neurons to the apex, driven by *Neurog1* [7,55]. Specific gene deletions eliminate SGNs. For example, *Pax2^Cre^*; *Gata3^f/f^* shows a small SGN that innervates the cochlear hair cells, while *Foxg1^Cre^*; *Gata3^f/f^* shows the absence of all SGNs and eliminates all cochlear hair cells [37]. An absence of SGNs is shown in *Shh* null [32] and *Lmx1a/b* DKO [38], while a small population of *Pax2* KO seems to form, but never reaches the usual position ventral to the brainstem [35]. A small population initially forms to develop SGNs but is lost in the cochlea before E14.5 [47]. There is increased neuronal formation, with abnormal extensions into the cochlea and brainstem [6,41].

**Figure 4:**
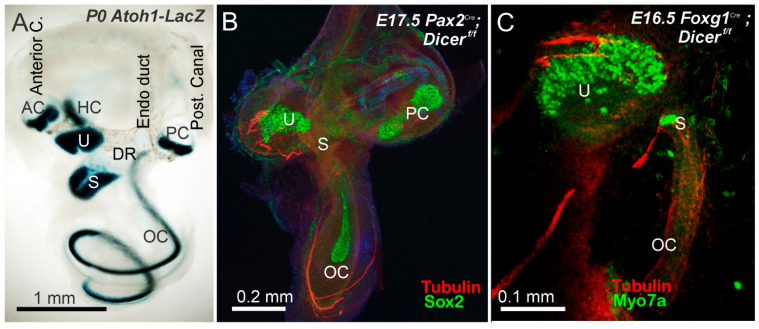
A comparison of control reduction with Dicer deletions. The five vestibular hair cells (AC, HC, U, S, PC) and the organ of Corti (OC) can be found using *Atoh1-LacZ* (**A**). A much smaller ear is seen in *Pax2^Cre^*; *Dicer^f/f^*, which shows only fibers reaching the utricle, saccule, and organ of Corti (**B**). Similarly, an even smaller utricle with very few fibers and a patch of the saccular hair cells can be found in *Foxg1^Cre^*; *Dicer^f/f^*. While few fibers extend along the small OC in both Dicer mutants, only one shows a small patch of cochlear hair cells (**B**,**C**). A PC without innervation is found in *Pax2^Cre^*; *Dicer^f/f^*, while all canal cristae are absent in *Foxg1^Cre^*; *Dicer^f/f^*. Abbreviations: AC, anterior crista; DR, ductus reuniens; HC, horizontal crista; OC, organ of Corti; PC, posterior crista; S, saccule; U, utricle. Modified from (**B**,**C**) [28,29] using antibody staining (**B**,**C**) or in situ hybridization. Sizes are shown on the bars.

**Figure 5:**
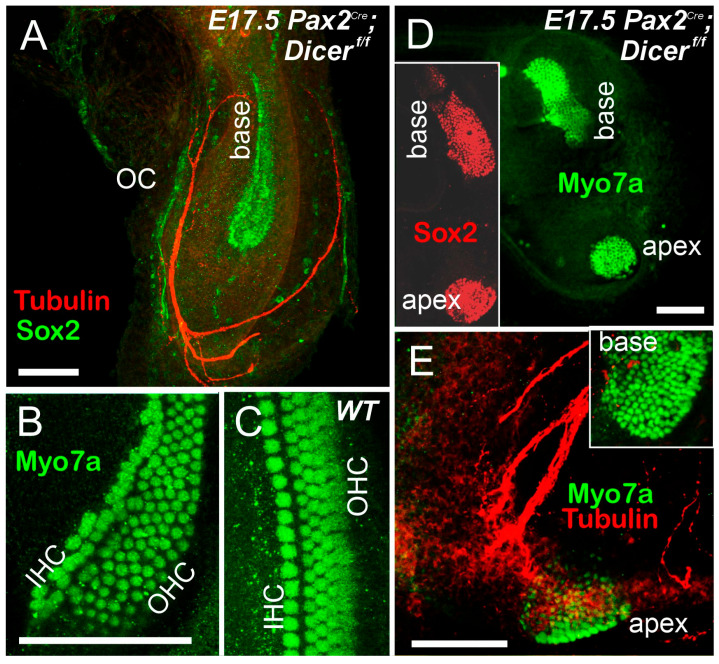
An organ of Corti develops in *Pax2^Cre^*; *Dicer^f/f^*. The organ of Corti (OC) can form either from a single population at the base (**A**) or be divided into two populations: a base and an apex [(**D**,**E**); inset (**D**,**E**)]. Antibodies against Myo7a reveal multiple rows of inner and outer hair cells in Dicer null mice, while control mice show one row of inner hair cells and three rows of outer hair cells (**B**,**C**). Fibers can be seen navigating around the apical region (**A**), potentially reaching the apex if present (**E**). Modified from (**B**) [29] with antibodies. Bar shows 100 µm.

In the absence of Dicer, no SGN develops (Figure 2, Figure 3 and Figure 4). It is dependent on *Pax2^Cre^*; *Dicer^f/f^* [29] or *Foxg1^Cre^*; *Dicer^f/f^* [28]. Dye tracing (Figure 1 and Figure 2) or using antibodies with tubulin shows a very few fibers that extend to the cochlear hair cells in *Pax2^Cre^*; *Dicer^f/f^*, but selective innervation is lost (Figure 4 and Figure 5). Likewise, very few fibers expand toward the cochlea [28]. In summary, without Dicer, there is nearly complete SGN loss, and the few fibers that remain are likely the inner ear efferent fibers (IEE; [56]).

### 2.3. Absence of Specific Hair Cells After Dicer Deletion

The sequence of events leading to the development of vestibular and cochlear hair cells starts with *Prox1* in the anterior and posterior canals at E11.5, which upregulates *Atoh1* to initiate vestibular hair cells formation, followed by a graded progression in the cochlea [4,57,58]. Specific null mutations show the loss of hair cells in the posterior crista (PC) [*Fgf10*; [50]] or horizontal crista (HC; *Foxg1*; [51]). In contrast, an expansion can ensue in the PC in *Lmx1a* null mice [59], and can be nearly eliminated in the anterior (AC) and horizontal cristae, while the posterior crista can be very large in *Lmx1/Lmx1b* DKO [38]. A normal AC in *Pax2^Cre^*; *Gata3^f/f^* mice show a reduced PC and HC. A typical AC forms in *Foxg1^Cre^*; *Dicer^f/f^*, but is absent in the HC with a tiny PC [37]. In contrast, only a PC develops in *Pax2^Cre^*; *Dicer^f/f^*, while all canal cristae and hair cells are absent in *Foxg1^Cre^*; *Dicer^f/f^*.

The otoconia-bearing utricle and saccule depend on genes that separate them into two populations of vestibular hair cells. *Otx1* [60], *Lmx1a* [59] or *Lmx1a/Lmo4* [61] deletion, resulting in utricular/saccular fusion. In *Irx3/4* DKO mice, there is a fusion between the base of the cochlea and the saccule [39]. Most important is the absence of tectorial membrane and otoconia that are likely absent in *Foxg1^Cre^*; *Dicer^f/f^*, but a small otoconia can be seen in the *Pax2^Cre^*; *Dicer^f/f^* mice (Supplement Figure S1; [28,29,30]). Hair cells develop in the utricle but have few vestibular hair cells (Figure 3). Moreover, a few fibers extend into the utricle, as shown with dye tracing (Figure 3A) or antibody labeling (Figure 3C and Figure 4B,C). In summary, utricular hair cells develop in either *Pax2* or *Foxg1^Cre^*; Dicer deletion, but show a reduced presence in a small saccule that also shows a dependence on *Neurog1* [7,33].

Cochlear hair cells develop into two populations: one row of inner hair cells (IHC) and three rows of outer hair cells (IHC and OHC; Figure 5C; [8,58]). Detailed analyses show a reduction in the base and loss of OHCs, while in the apex, the number of OHCs increases and has many hair cells compared to IHCs [4]. A shorter cochlea is shown in *Neurog1* [33], *Foxg1* [51], COUP-TF1 [62], *Insm1* and *Tbx2* [63] or *MycN* null deletions [64]. In particular, the apex expands its rows in three mutants [33,51,64], while the shorter base is near normal with one row of IHC and three rows of OHCs. Monotremes, a basic mammal, have expanded IHCs and OHCs and have a unique population in the apex, and the lagena [65,66]. *Pax2^Cre^*; *Gata3^f/f^* shows patches of hair cells [37]. Likewise, *Fgfr1* shows patches of cochlear hair cells [67], which also shows hair cell patches in *Sox2^Ysb/Ysb^* mice [68,69]. An absence of cochlear hair cells is shown in *Foxg1^Cre^*; *Gata3^f/f^*, *Lmx1a/b* DKO [38], and an unusual *Pax2* cochlear nerve underneath the brainstem that is devoid of cochlear hair cells [35].

Compared to cochlear hair cells, if present at all, they show a form of two outcomes: either a shorter, increasingly wider form or a split shorter basal turn that separates from the apex in *Pax2^Cre^*; *Dicer^f/f^* (Figure 5A,B,D,E; [29]). Hair cells can split into the IHC and the much broader OHC, or do not show any distinction from IHC/OHC compared to control (Figure 4B,C). Fibers are expanding, likely from IEE, and receive innervation (Figure 5A,E). *Foxg1^Cre^*; *Dicer^f/f^* lacks all cochlear hair cells [28]. In summary, one conditional deletion of Dicer results in a single or two patches of cochlear hair cells (*Pax2^Cre^*; *Gata3^f/f^* or *Pax2^Cre^*; *Dicer^f/f^* that resemble monotreme organization (base/middle; distinct apex), while *Foxg1^Cre^*; *Dicer^f/f^* resembles *Foxg1^Cre^*; *Gata3^f/f^* conditional deletions of all cochlear hair cells, resembling *Shh* KO, *Lmx1a/b* DKO and *Pax2* null mice [32,35,38,70].

### 2.4. Vestibular Fibers Reach out to the Caudal Brainstem but Never Innervate the Cerebellum

**Figure 6:**
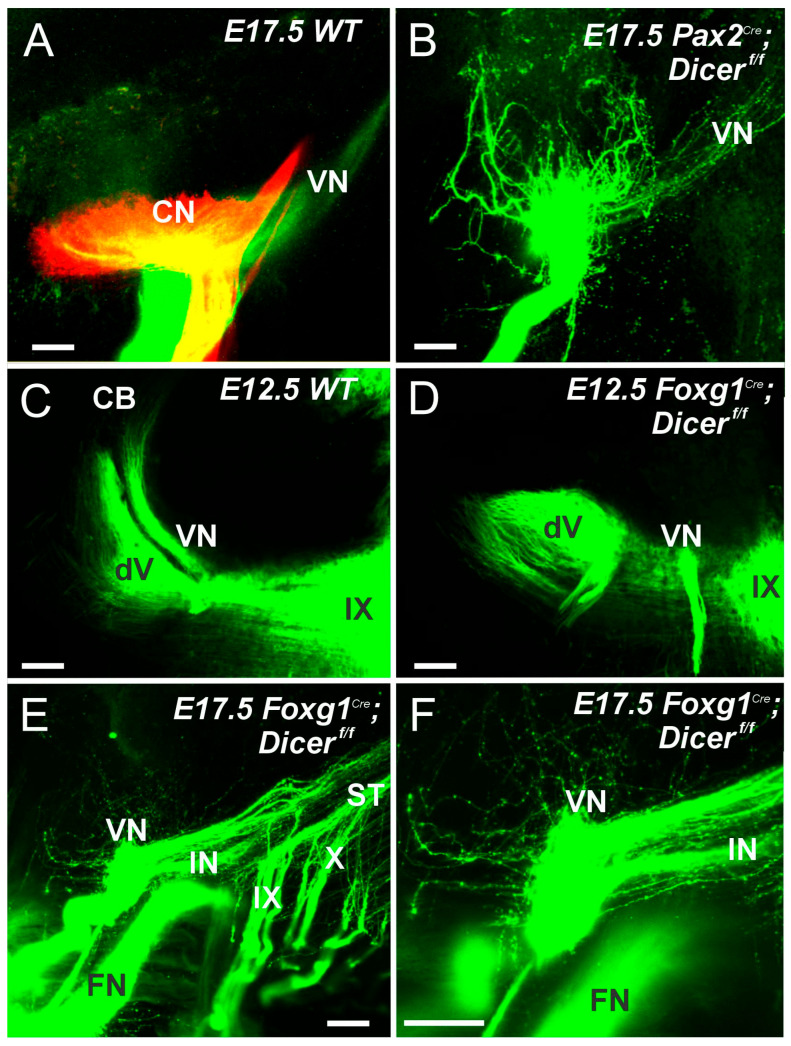
Dye tracing shows the nearly absent central projection in Dicer null mice. (**A**) A control mouse displays cochlear fibers in red and vestibular fibers in green. Note that the cochlear nuclei (CN) extend from r2–5, while a larger caudal projection is seen in the vestibular nuclei (VN). The projection of both cochlear and vestibular fibers from a *Pax2^Cre^*; *Dicer^f/f^* mouse shows a ramification across the dorsal rhombomeres and shows a caudal projection from the VN (**B**). Unlike *Pax2^Cre^*; *Dicer^f/f^* mice show no rostral projections reaching the cerebellum (**C**,**D**), which projects typically parallel to the trigeminal fibers (dV) in control mice. The central projection runs parallel to the inferior nerve (IN), which arises from neurons in the geniculate ganglion. (**E**,**F**) The entry point of the facial nerve (FN) is near the IX fibers. Note the few fibers that extend more rostrally and dorsally, showing a caudal projection next to the IN fibers. Abbreviations: CN, cochlear nuclei; FN, facial nerve; IN, intermediate nerve; VN, vestibular nuclei and fibers; IX, glossopharyngeal nerve; X, vagus nerve. Modified after (**A**,**B**) [29] using lipophilic dye tracing. The bar shows 100 µm.

A distinct cell population generates *Atoh1* to produce cochlear nuclei (CN), which also gives rise to a more ventral population of neurons. In *Ptf1a* null mice, neurons migrate to the CN [71,72]. Interestingly, even if *Atoh1* is deleted in all CN and all HC, the central projection shows the same topology as usual, which likely receives the *Ptf1a* nuclei [73,74]. In contrast to the elementary organization of *Atoh1*/*Ptf1a*, the vestibular nuclei are more complex and spread much more widely [8,75,76]. While the CN consists of only four nuclei (rhombomeres (r) 2–5), the VN extends from the cerebellum to r9. Selective deletion using specific deletion of *Atoh1* can show the unique absence of specific rhombomeres that alter the central projection [40]. Central projection can be a misrouted projection in *Gata3*, *Neurod1* or *Isl1* conditional deletions [6,37,41]. In addition, using the conditional deletion of Dicer showed a reduction in neurons in the brainstem [31]. *Pax2^Cre^*; *Dicer^f/f^* have a broad expression in the midbrain, the brainstem, and the ear, and eliminate the cerebellum and much of the brainstem with the Dicer deletion [29]. In contrast, *Foxg1^Cre^*; *Dicer^f/f^* is highly expressed in the forebrain, the anterior part of the eye, the cerebellum, and the ear [28]. A significant difference in Dicer expression should be seen in the brainstem between *Pax2* and *Foxg1*. Results show a central projection of control mice labeled from dye tracing of cochlear and vestibular fibers (Figure 6A). In contrast, central projections of *Pax2^Cre^*; *Dicer^f/f^* are ramified across CN fibers that do not show a tonotopic organization, do not reach the cerebellum, and show a caudal projection of VN (Figure 6B). The ramifications of fibers in the absence of SGN are still unclear. Central projection of *Foxg1^Cre^*; *Dicer^f/f^* shows only a minimal projection that exclusively innervates caudal vestibular fibers. Compared to the central projection in E12.5, old mice show projections in parallel to the trigeminal fibers (Figure 6C), while no segregation of trigeminal and vestibular projections is shown in *Foxg1^Cre^*; *Dicer^f/f^* (Figure 6D). A closer examination of the inner ear fibers with the intermediate nerve and facial nerve shows that all remaining central projections expand exclusively to the caudal rhombomere 4–9 (Figure 6E,F), which shows a tiny projection around the CN. In summary, central projection of cochlear and vestibular fibers can show segregation, while in *Pax2^Cre^*; *Dicer^f/f^*, there is no sign of a topology and ramifying fibers around the CN and follow caudal VN. In contrast, in *Foxg1^Cre^*; *Dicer^f/f^*, they have only very few fibers ramifying across the CN and have a caudal projection next to intermediate nerves to innervate the taste buds.

## 3. Discussion

Dicer is a central protein that allows the conversion of pre-miRNAs into miRNAs [77,78,79,80]. Despite the central function of Dicer [16], other miRNAs can further upregulate after Dicer deletion [20,81]. In addition, after conditional deletion of Dicer, miRNA can be expressed for hours and days, which will diminish with the presence of miRNA [29]. Given that over 100 miRNAs have been partially identified for the ear [9]. In contrast, the total miRNAs can vastly increase beyond the current identified ~500 miRNAs [16], which leaves open how many miRNAs survive longer or shorter after Dicer deletion. With this warning in mind, we aim to provide an overview of two conditional deletions of Dicer using *Pax2^Cre^* and *Foxg1^Cre^* for conditional deletion of Dicer.

### 3.1. The Absence of Dicer Results in the Loss of Nearly All Ear Neurons

Nearly all vestibular neurons show a rapid loss of early expression (Figure 1 and Figure 2) that disappears in miR-124 [29]. Spiral ganglion neurons develop in either *Pax2^Cre^* or *Foxg1^Cre^* with Dicer deletion. We know that the earliest vestibular neurons develop at about E9 [33,42], while SGNs develop later at approximately E10 [42,55]. This progression of Dicer deletion fits with *Foxg1^Cre^*; *Sox2^f/f^*, which initially develop near-normal, but do not expand to innervate the cochlear hair cells [47], or *Foxg1^Cre^*; *Gata3^f/f^* eliminates all SGNs [37]. Deletion is more effective in eliminating all SGNs in *Lmx1a/b* DKO mice [38] or *Pax2* KO [35]. The remaining vestibular neurons in older mice show the innervation of the utricle (Figure 3 and Figure 4) consistent with other vestibular deletions. Dye insertion and tubulin antibody labeling, combined with caspase 3 expression, document the loss of nearly all peripheral inner ear neurons [28,29,30]. The survival of specific small vestibular neurons after Dicer deletion can be attributed to several potential factors:

1. Cell-Specific Functions of Dicer: Certain vestibular neurons may have a distinct set of survival pathways or compensatory mechanisms that allow them to thrive despite the loss of Dicer.
2. Differential Expression of miRNAs: The small vestibular neurons innervating the utricle may be less dependent on specific microRNAs for their survival compared to other neurons. They may express distinct sets of miRNAs that confer resilience to the effects of Dicer deletion.
3. Regional Differences: The utricle may provide unique environmental factors or signaling cues that promote the survival of these specific neurons, such as growth factors or extracellular matrix components that are not present in other areas of the vestibular system, such as *Bdnf* and *Ntf3* [82].
4. Developmental Timing: The timing of Dicer depletion may influence neuron survival. If Dicer is removed at a stage where specific small vestibular neurons have already formed critical connections or survival mechanisms, they might tolerate the loss better than others.
5. Intrinsic Neuronal Properties: The intrinsic properties of these neurons, such as their metabolic demands or signaling pathways, could make them more resilient to stress induced by the lack of miRNA regulation.

Further work is needed to clarify the survival of a very few vestibular neurons that innervate the utricle nearly exclusively.

### 3.2. Absence of Dicer Results in Differential Loss of Vestibular and Cochlear Hair Cells

Hair cell development starts in the anterior and posterior crista, where *Prox1* is expressed, followed by *Sox2* and *Atoh1* for vestibular and cochlear hair cells [57,83,84,85]. Three distinct hair cells form: the canal cristae respond to angular acceleration, the utricle and saccule respond to linear acceleration and gravity, and the cochlea responds to different frequencies for hearing. Certain deletions are known, such as specific loss of posterior crista in *Fgf10* or horizontal crista in *Foxg1* null mice [50,51], while other deletion shows the absence of cochlear hair cells in *Lmx1a/b* DKO, *Pax2* KO or *Foxg1^Cre^*; *Gata3 ^f/f^* mice [35,37,38,86].

**Figure 7:**
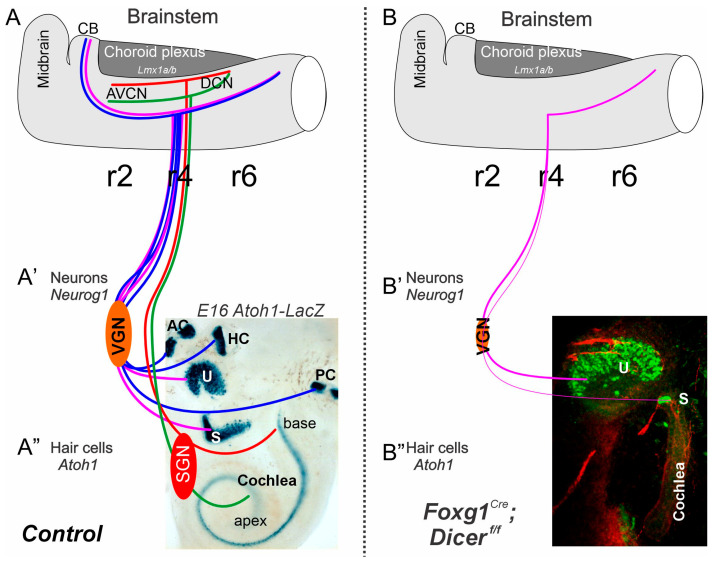
Comparison of control and *Foxg1^Cre^*; *Dicer^f/f^*. (**A**) Two neurons (VGN, SGN; **A′**) reach out to innervate the five vestibular hair cells and the cochlea (**A″**). Central projection is split into the cochlear nuclei that extend from r2–5 and have a topological input from the base and the apex (**A″**; red, green). Vestibular fibers stretch from the cerebellum (CB) to r8/9. The color is shown in utricle/saccule (lilac) versus canal cristae (blue). (**B–B″**) *Foxg1^Cre^*; *Dicer^f/f^* has no cochlear neurons or hair cells, no canal cristae, but has a small VGN with a small utricle and an even smaller saccule. Central projection reaches out toward caudal, not rostral to the cerebellum.

Compared to the two Dicer conditional deletions, a difference is noticeable: only the posterior crista forms in *Pax2^Cre^*:*Dicer^f/f^*, while all canal cristae are absent in *Foxg1^Cre^*; *Dicer^f/f^*. The presence of the posterior crista can likely depend on differential expression of *Pax2*, which has the same effect as *Pax2^Cre^*; *Gata3^f/f^* [37,87] compared to *Foxg1^Cre^*; *Gata3^f/f^* [37]. It shows nearly complete loss of the posterior crista. It is unclear why the utricle stays and has vestibular hair cells while the nearby saccule is reduced to a few hair cells (Figure 3, Figure 4 and Figure 7). As pointed out on the survival of a very few vestibular neurons, the same arguments can be raised for the few surviving vestibular hair cells in the utricle (and posterior crista).

A distinction of cochlear hair cells shows nicely in the comparison (Figure 4): *Pax2^Cre^*; *Dicer^f/f^* has formed cochlear hair cells, while *Foxg1^Cre^*; *Dicer^f/f^* has no cochlear hair cells (Figure 4, Figure 5 and Figure 7). A similar effect is in *Pax2^Cre^*; *Gata3^f/f^* shows a patch of neurons, while *Foxg1^Cre^* eliminates all cochlear hair cells, which is likely related to the slight progression of conditional deletions [37]. Additional information that regulates *Neurod1*, *Notch*, *Jag1*, *Hes*, and others play a role in abnormal cochlear patterning, increased sensory proliferation, and extra inner and outer hair cells [42,51,62,63,88,89,90] that requires a deeper understanding of the results of Dicer deletion. What is most interesting is the split into two populations in Gata3 or Dicer conditional deletion using *Pax2^Cre^*: a split into base/middle and an apex (Figure 5 and Figure 7). In addition, a base/middle and apex are shown in *MycN* null mice [64], while patches of hair cells are documented in *Fgfr1* deletion [67] or *Sox2^Ysb/Ysb^* deletions [68]. Whether or not this apex patch is compared to the lagena in monotremes remains to be seen [4,65].

### 3.3. The Absence of Rostral Projection Shows Fibers Toward the Caudal Brainstem

Central projection receives a distinct tonotopic organization from the SGN to innervate the cochlear nuclei (CN) of rhombomere 2–5 (Figure 7; [1]), while vestibular projection, without a topological organization, spreads from the cerebellum to the caudal tip of rhombomere 9 to innervate vestibular nuclei [4,42,76]. Specific deletions of *Neurod1*, *Isl1,* and *Gata3* show a ramification to innervate the CN, [6,37,41,91], even if certain nuclei are absent in the conditional deletion of specific rhombomeres of *Atoh1* [40]. Even a near-normal topology can happen without the CN and hair cell development [73,74]. Moreover, in the absence of *Lmx1a/b*, DKO mice show no SGNs, no cochlear hair cells, and no cochlear nuclei showing branches of vestibular fibers to ramify across the cerebellum and cross to the contralateral side [38].

*Pax2^Cre^* or *Foxg1^Cre^*, after Dicer deletion, should be differentially affected in the brainstem (Figure 6). *Pax2* is broadly expressed in the cerebellum, the ear, and the brainstem, while *Foxg1* is expressed in the forebrain, part of the eyes, the ears, and the cerebellum [28,29]. Another conditional deletion of Dicer using *Egr2^Cre^* shows a reduced volume formation of the cochlear nuclei. It reduces specific SOCs, whereas delayed upregulation of *Atoh1^Cre^* via Dicer deletion results in near-normal cochlear nuclei [31]. In the absence of SGNs, we show a different central projection that ramifies across the CN in *Pax2^Cre^*; *Dicer^f/f^* (Figure 6; [29]) and shows a comparable ramification that has enough SGNs in *Pax2^Cre^*; *Gata3^f/f^* to innervate the CN [37]. Most interesting is the central projection in *Foxg1^Cre^*; *Dicer^f/f^* that lacks all cochlear nuclei and cochlear hair cells and has a small population of VGN to reach out to the VN (Figure 6E,F and Figure 7). Nearly all fibers reach out toward rhombomere 4–9 and show a very few branches that ramify around the CN. It is possible that, in VGN and the small population of the utricle, projection is only caudal, while in normal central projection, it splits into rostral and caudal neurons that show a unique pattern of the utricle [92,93,94]. Moreover, the polarity is different in the utricle between the fibers innervated from the cerebellum versus the caudal brainstem. Unfortunately, conditional deletion does not survive beyond birth in postnatal mice, preventing further analysis of the remaining fibers and their peripheral and central projections and functions [93].

## 4. Materials and Methods

The absence of Dicer results in early embryonic lethality, but the use of *Dicer^f/f^* allows examination of later deletions [27]. We employed conditional deletion of Dicer to show the loss of later expression of two genes, *Pax2^Cre^* and *Foxg1^Cre^* [95,96], to eliminate Dicer expression (*Pax2^Cre^*; *Dicer^f^^/f^* and *Foxg1^Cre^*; *Dicer^f^^/f^*). Embryos were collected from pregnant dams on embryonic days (E) 11.5, 12.5, 14.5, 16.5, and 18.5 that would be dead before birth. There is no replacement for animals, and the minimal number of mice was used. Moreover, mice were housed in a 12/12 cycle provided with food and water in an enriched environment. All animal care and procedures were reviewed by an ethics committee and approved by the University of Iowa Institutional Animal Care and Use Committee (IACUC; #0021971; 19 February 2020).

Pregnant dams were anesthetized with 1.25% Avertin (tribromoethanol) solution at a dose of 0.025 mL/g by IP injection. Deeply anesthetized mice were not responding to pinches. Mice were euthanized after being deeply anesthetized, free of pain, suffering, and distress. Both male and female embryos were used. Embryos were dissected from the uterus and perfusion fixed with 4% PFA in 0.1M phosphate buffer (pH 7.4) with 300 mM sucrose [97]. We used about three Dicer deletion mice of each *Pax2* and *Foxg1* null and control mice spanning the range of E11.5–E18.5. The smaller size of the ears makes it difficult to dissect the ears, especially in the smallest *Foxg1^Cre^*; *Dicer^f^^/f^*. Given the small ears, we used them as whole mounts to see the 3D organization and the remaining innervation.

Immunostaining: We used immunostaining to show specific information. Briefly, antibodies were used to show the nerve fibers with tubulin (SigmaAldrich, St. Louis, MO, USA; anti-α-tubulin #T5168; the RRID–AB_477579 ratio; dilution: 1:500) and show the organization of hair cells using Myo7a (Proteintech, Rosemont, IL, USA; anti-Myo7a #83807; the RRID–AB_3671394 ratio; dilution: 1:700) and Sox2 (Biotechn, Mumbai, India; anti-Sox2 #AF2018; the RRID–AB_355110 ratio; dilution: 1:1000). Tissues were fixed with 4% PFA in 0.1 M phosphate buffer (pH 7.4) and defatted with ethanol, blocked with goat serum with 0.5% Triton X-100, and incubated for one day with primary antibody. Secondary antibody (Thermo Fischer Scientific, Waltham, MA, USA) was conjugated to either Alexa 488, Alexa 543 or Alexa 648 using a dilution of 1:500. Tissues were mounted in glycerol and viewed using a Zeiss confocal microscope (Oberkochen, Germany). In addition, we used *Atoh1^LacZ^* to label hair cells [7].

Dye tracing: We used lipophilic dye tracing to label fibers and neurons from brainstem dye insertion to the ear and vice versa [39,97]. The ears were used to enable whole-mount imaging in younger mice, while tissue from older mice was prepared to visualize the ganglia and their innervation to the hair cells. Central projections were split at the midline and flattened to display fiber input. In the oldest mice of *Foxg1^Cre^*; *Dicer^f/f^*, which were too small, we inserted dyes to label the VII, VIII, and IX nerves. Images were captured with a confocal system, either (Jena, Germany), using 10× and 20× objectives (NA 0.4 or 0.8). The images were compiled into plates using CorelDraw, 2025.

## 5. Conclusions

Dicer is essential for the development of miRNA in the brain, eyes, ears, and other body parts. Using conditional deletion of Dicer in specific tissues allows at least the earliest stages of ear development and the formation of its central projections. Despite Dicer deletion, some vestibular ganglion neurons stay, and there is still innervation of vestibular hair cells within the utricle. Although cochlear hair cells do not show innervation, their development follows a pattern of segregation into basal/middle and apex regions. This suggests that the level of developmental organization persists despite the absence of Dicer. Projections from the utricle mainly target the caudal brainstem. These fibers may innervate vestibular hair cells exclusively at specific polarities, showing specialization in neural connections during development.

The findings highlight the complexity of ear and neural development, showing that specific structures can still form and function even with significant genetic changes. Currently, we understand the role of Dicer in LUCA [25,26] and the genetics of vertebrate ears [1,2,3,42] that show the role of miRNA [9,12,14,28,29]. Further research could reveal new pathways and therapeutic targets for treating hearing and balance disorders related to miRNA regulation.

## Data Availability

The original contributions presented in this study are included in the article/Supplementary Material. Further inquiries can be directed to the corresponding author.

## Supplementary Materials

The following supporting information can be downloaded at: https://www.mdpi.com/article/10.3390/ijms27010539/s1.
