# Supplementary material for: Dicer Deletion in the Ear Can Cut Most Neurons and Their Innervation of Hair Cells to Project to the Ear and the Brainstem
Source: Int J Mol Sci. 2026 Jan 5;27(1):539. doi: 10.3390/ijms27010539 (PMC12787206; doi:10.3390/ijms27010539)
Supplement: Supplementary file 1 [file ijms-27-00539-s001.zip › ijms-4061948-supplementary.pdf]

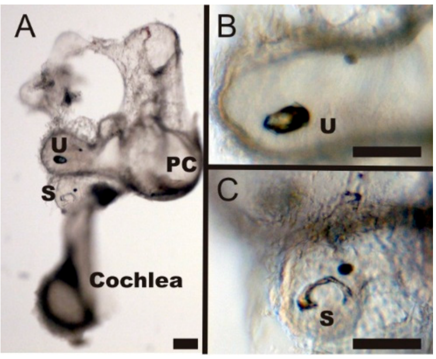

Figure S1. The defects on ear morphogenesis and histogenesis of a conditional Dicer null are shown for E17.5 *Pax2* ^{Cre}; *Dicer* $^{f/f}$ null mouse. The ear invariably lacks a recognizable anterior and horizontal crista but shows a well-developed posterior crista and canal (PC; A). Occasionally (2 of 8 ears), a partial anterior canal form, consisting of vesicles interconnected by a strand (A). There is no formation of a horizontal canal, and a small saccule is found only rarely (4 of 8 ears). The utricle is a simple sack that has a single otolith (B), and the saccule may have a small misshapen otoconium (C). Published [A-C; [19]. Bar shows 100 μm.
